# Bilateral synchronous papillary renal neoplasm with reverse polarity and renal cell carcinoma with fibromyomatous stroma: a case report and review of the literature

**DOI:** 10.3389/fonc.2025.1668258

**Published:** 2025-11-24

**Authors:** Guiwu Chen, Xiaomin Liao, Wenqin Liu, Jiaxin Meng, Yuting Li, Xiaoling Leng

**Affiliations:** 1 Department of Ultrasound, The Tenth Affiliated Hospital, Southern Medical University, Dongguan People’s Hospital, Dongguan, China; 2 Department of Pathology, The Tenth Affiliated Hospital, Southern Medical University, Dongguan People’s Hospital, Dongguan, China; 3 Department of Radiology, The Tenth Affiliated Hospital, Southern Medical University, Dongguan People’s Hospital, Dongguan, China

**Keywords:** renal tumors, synchronous tumors, papillary renal neoplasm with reverse polarity, renal cell carcinoma with fibromyomatous stroma, ultrasound, computed tomography, pathology

## Abstract

**Background:**

This report presents an exceptionally rare case of bilateral synchronous renal tumors comprising papillary renal neoplasm with reverse polarity (PRNRP) and renal cell carcinoma with fibromyomatous stroma (RCC-FMS) in a single patient. No prior cases of this specific combination occurring synchronously and bilaterally have been reported.

**Case presentation:**

A 65-year-old man presented with incidentally detected bilateral renal masses. Abdominal ultrasound and contrast-enhanced computed tomography (CT) revealed distinct imaging characteristics for each tumor. The right kidney mass was exophytic, heterogeneous, and hypovascular on ultrasound, showing marked heterogeneous enhancement with hypoenhancing foci on CT. The left kidney mass was a well-circumscribed, mixed-attenuation nodule with peripheral/septal enhancement on CT. The patient underwent bilateral laparoscopic partial nephrectomy. Histopathological and immunohistochemical analysis confirmed PRNRP in the right kidney (CK7+, GATA3+, Ki-67 approximately 2%) and RCC-FMS in the left kidney (PAX-8+, CA IX+, CD10+, Ki-67 approximately 3%). Real-time quantitative-PCR testing was positive for a KRAS exon 2 mutation, but was negative for NRAS (exons 2-4) and BRAF V600 (exon 15) mutations.

**Conclusion:**

This represents the first documented case of synchronous bilateral occurrence of PRNRP and RCC-FMS. It highlights significant diagnostic challenges due to overlapping imaging features with more common renal tumors. It underscores the critical role of multimodal imaging (ultrasound, CT) combined with meticulous histopathology, immunohistochemistry, and molecular genetic analysis for accurate diagnosis. The generally indolent nature of both tumors supported successful nephron-sparing surgical management. This unique case emphasizes the need for a high index of suspicion for rare tumor subtypes and a multidisciplinary approach to optimize the diagnosis and tailored treatment of complex renal masses.

## 1 Introduction

Papillary renal neoplasm with reverse polarity (PRNRP), a rare renal tumor, is characterized by apical nuclear positioning, eosinophilic cytoplasm, and frequent KRAS mutations. Typically appearing as small incidental nodules with right kidney predominance, it exhibits indolent behavior without reported metastases (1). In contrast, renal cell carcinoma with fibromyomatous stroma (RCC-FMS) features clear cell epithelia embedded in smooth muscle stroma and mTOR pathway alterations (2). Though most cases are organ-confined, rare lymph node metastases occur in tuberous sclerosis complex-associated variants (3).

To our knowledge, there are no prior reports of bilateral synchronous rare renal tumors: PRNRP and RCC-FMS, which present significant diagnostic challenges due to their rarity and the need to differentiate them from more common mediastinal tumors. In this study, we present an extremely rare case of Bilateral Synchronous Rare Renal Tumors: PRNRP and RCC-FMS, which were characterized by ultrasound, computed tomography, and confirmed by pathology.

## 2 Case presentation

A 65-year-old man was referred to our hospital following the detection of bilateral renal masses during an evaluation at a community hospital two weeks prior. His medical history included laparoscopic left adrenal adenoma resection more than ten years ago and a four-year history of coronary heart disease status post cardiac stent placement. He was currently on standard medical therapy, including nifedipine, benazepril, bisoprolol, and atorvastatin.

**Figure 1:**
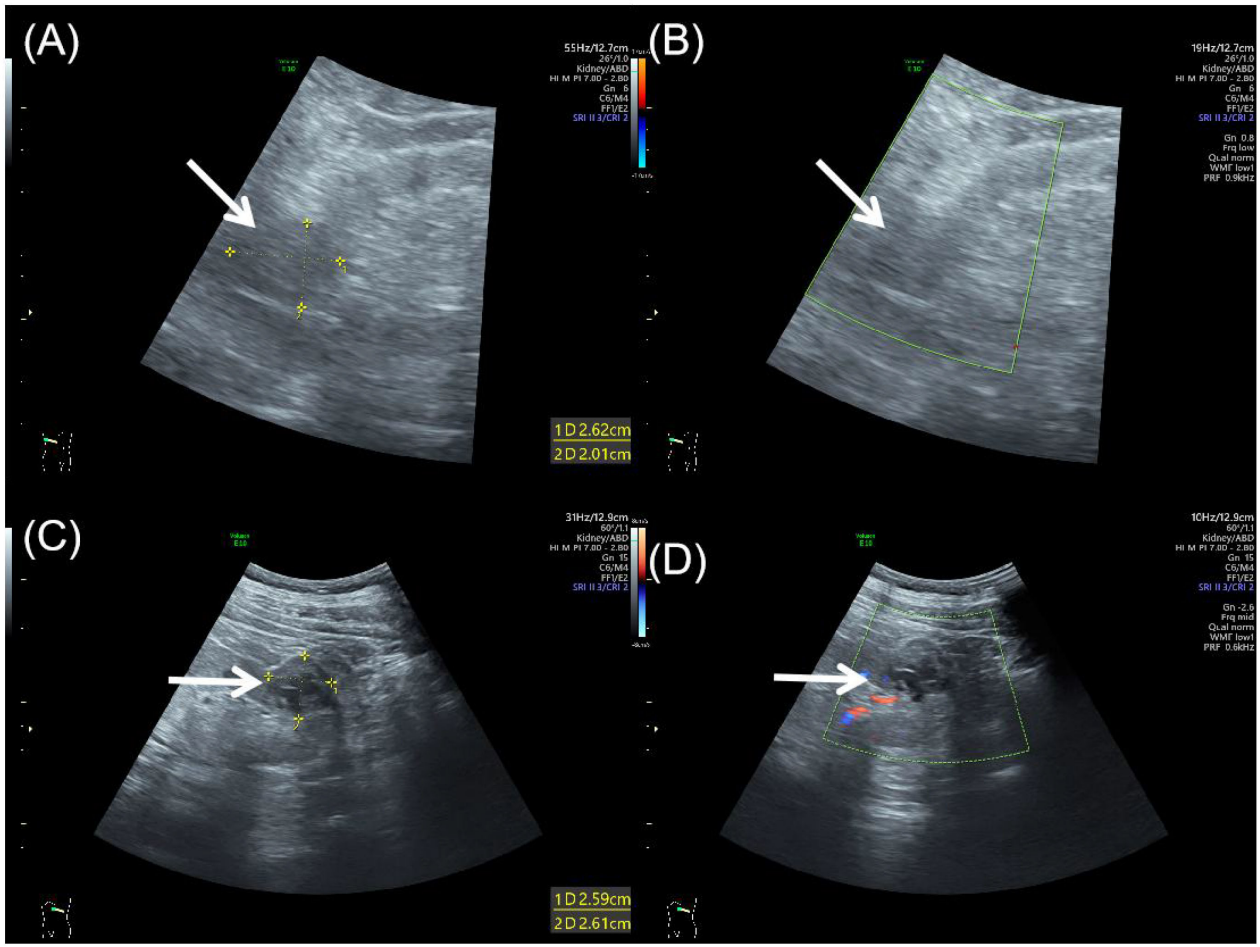
Abdominal ultrasound of bilateral synchronous rare renal tumors. **(A)** Grayscale ultrasound showed an oval, well-defined, heterogeneous mass (arrow) in the right kidney. **(B)** Color Doppler flow imaging showed no significant blood flow within the right renal mass (arrow). **(C)** Grayscale ultrasound showed a well-defined, heterogeneous cystic-solid mass (arrow) in the left kidney, with solid predominance and irregular hypoechoic areas. **(D)** Color Doppler flow imaging showed no significant vascularity in the left renal mass (arrow).

Abdominal ultrasound demonstrated bilateral renal masses, with the right kidney showing an oval, well-defined, and heterogeneous mass (**Figure 1A**) without significant internal blood flow (**Figure 1B**). A well-defined, regular, heterogeneous cystic-solid mass with solid predominance was observed in the left kidney (**Figure 1C**), showing irregular hypoechoic areas without significant internal vascularity (**Figure 1D**).

**Figure 2:**
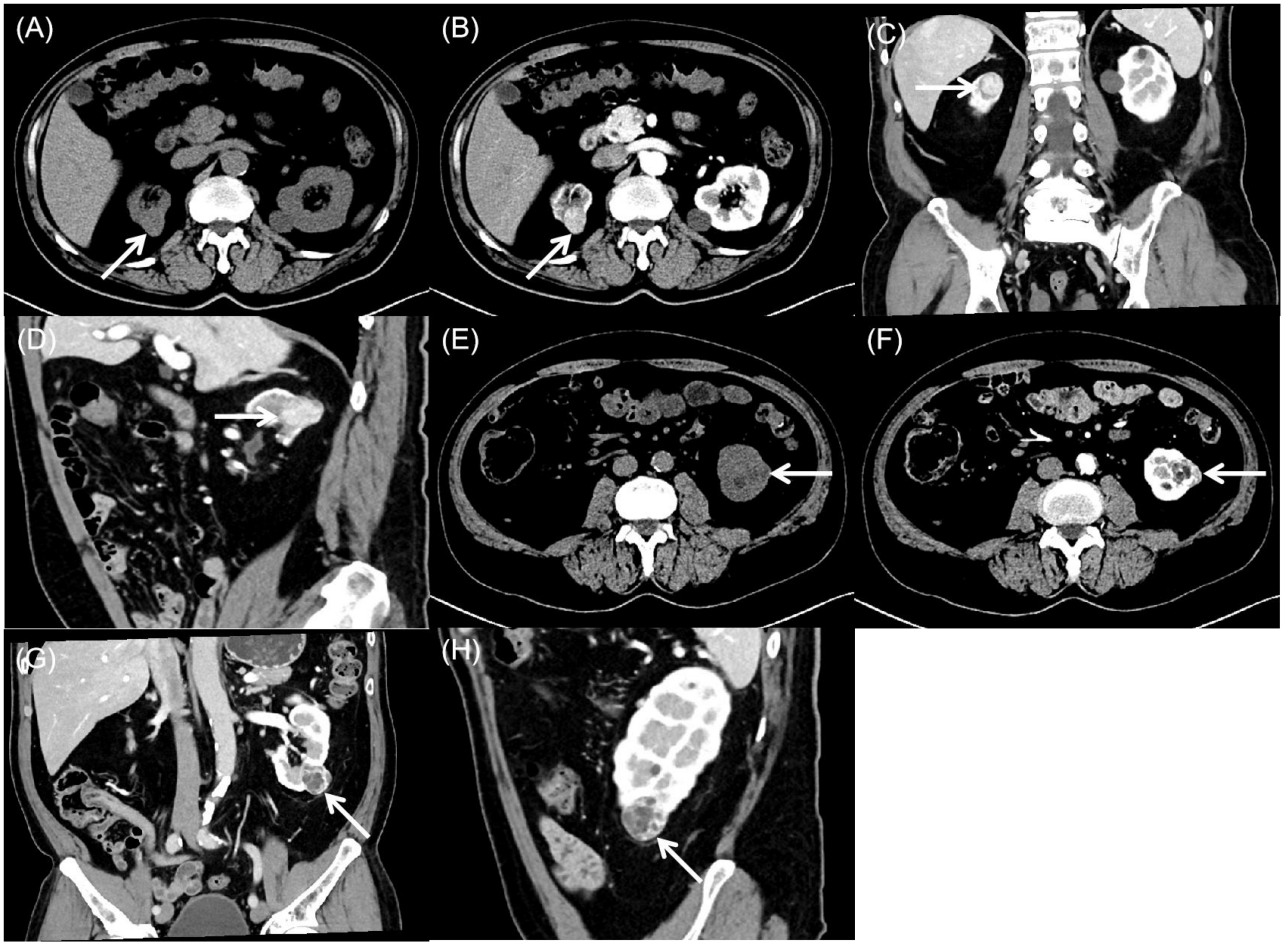
Computed tomography of bilateral synchronous rare renal tumors. **(A)** Plain computed tomography showed an exophytic, round, heterogeneous soft-tissue attenuation mass (arrow) in the right kidney. **(B-D)** Contrast-enhanced computed tomography showed marked heterogeneous enhancement during the cortical and medullary phases, with round hypoenhancing foci. Enhancement decreases in the excretory phase while the lesion maintains well-defined margins and persistent internal heterogeneity (arrow). **(E)** Plain computed tomography showed a well-circumscribed, heterogeneous mass (arrow) with mixed attenuation in the left kidney. **(F-H)** Contrast-enhanced computed tomography showed characteristic peripheral and septal enhancement, with progressive contrast retention in the delayed phase (arrow).

Computed tomography demonstrated bilateral renal nodules with distinct imaging characteristics. The right kidney exhibited an exophytic, roundish soft-tissue attenuation nodule that appeared heterogeneous on unenhanced images (**Figure 2A**). Following contrast administration, the lesion showed marked heterogeneous enhancement during cortical and medullary phases, containing roundish hypoenhancing foci, with subsequent decreased enhancement during excretory phase while maintaining well-defined margins and persistent internal heterogeneity (**Figures 2B–D**). Meanwhile, the left kidney lower pole contained a well-circumscribed, mixed-attenuation nodule (**Figure 2E**) demonstrating internal heterogeneity and characteristic peripheral and septal enhancement post-contrast (**Figures 2F–H**).

**Figure 3:**
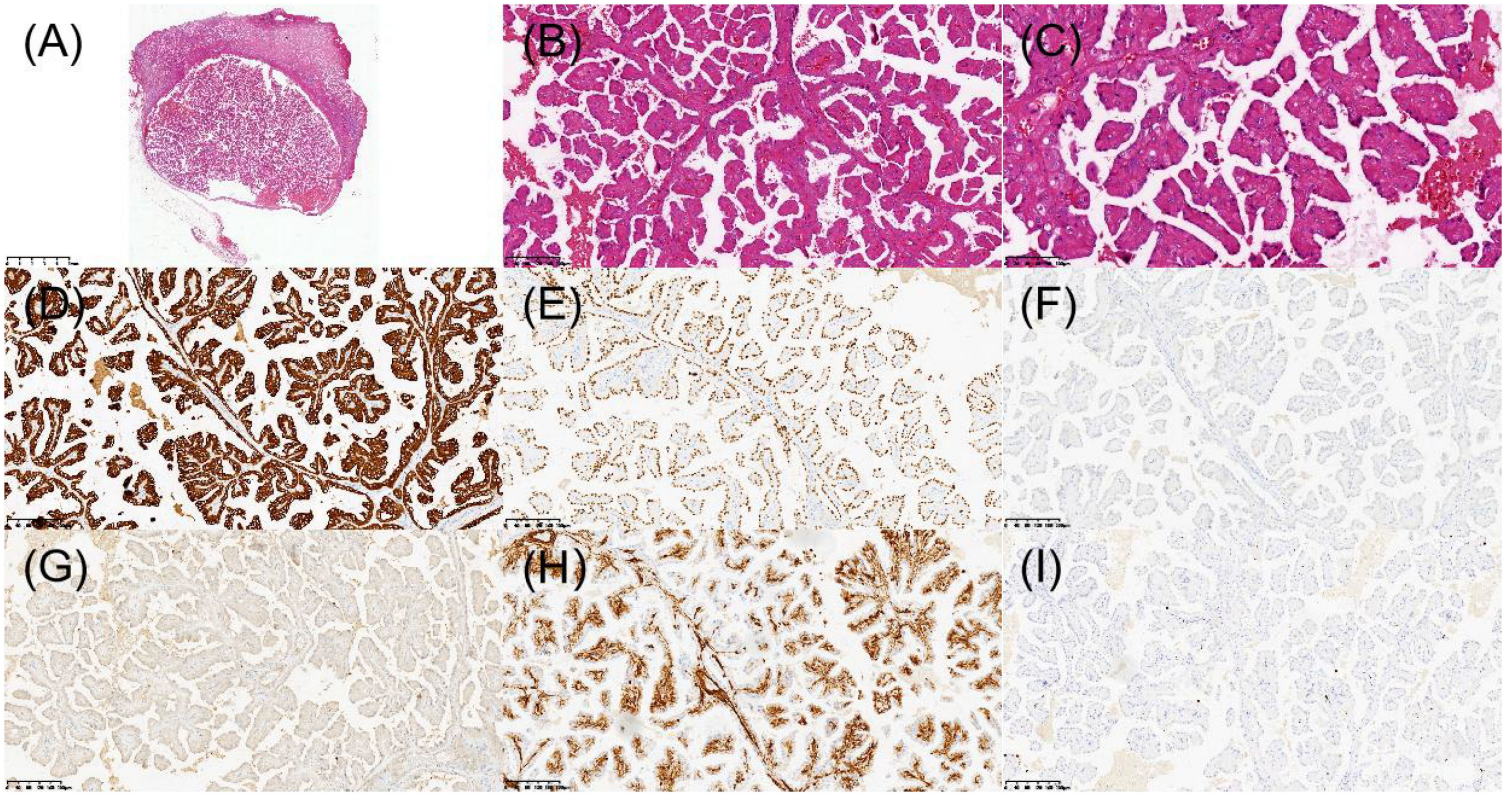
Histopathologic and immunohistochemical features of papillary renal neoplasm with reverse polarity in the right kidney. **(A)** H&E staining revealed a well-demarcated tumor under low-power microscopy. **(B)** Papillary architecture of the tumor. **(C)** Characteristic reverse nuclear polarity with apical positioning of nuclei. **(D)** Immunohistochemical staining demonstrated diffuse CK7 positivity. **(E)** Strong nuclear expression of GATA3. **(F)** AMACR was negative. **(G)** CD10 was negative. **(H)** Vimentin was positive in scattered individual cells. **(I)** Ki-67 was approximately 2%.

**Figure 4:**
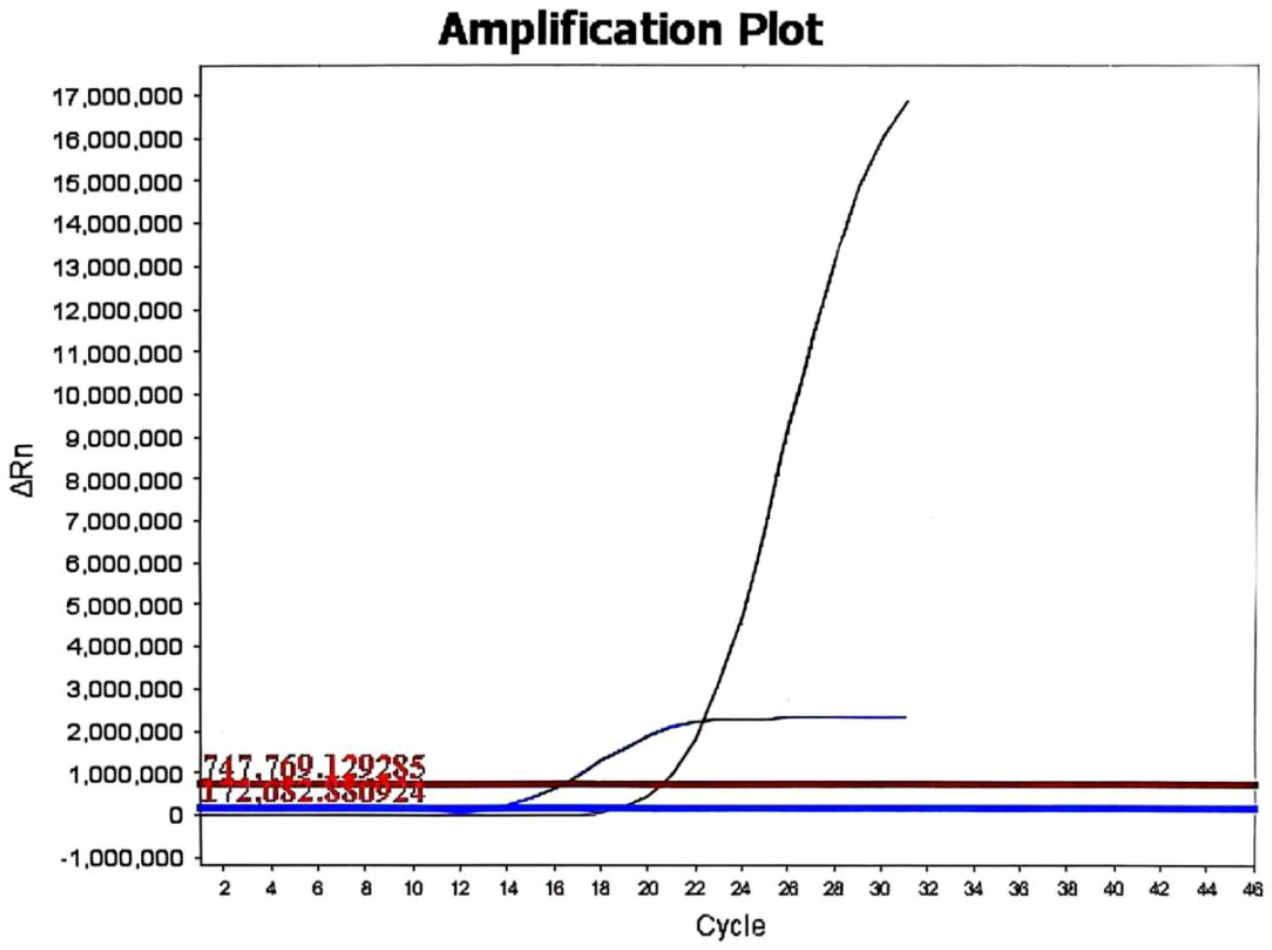
The KRAS mutation status of the papillary renal neoplasm with reverse polarity. Real-time fluorescent quantitative PCR analysis revealed a KRAS mutation in exon 2 (G12C, G12R, G12V, G12A, or G13C) in the papillary renal neoplasm with reverse polarity.

**Figure 5:**
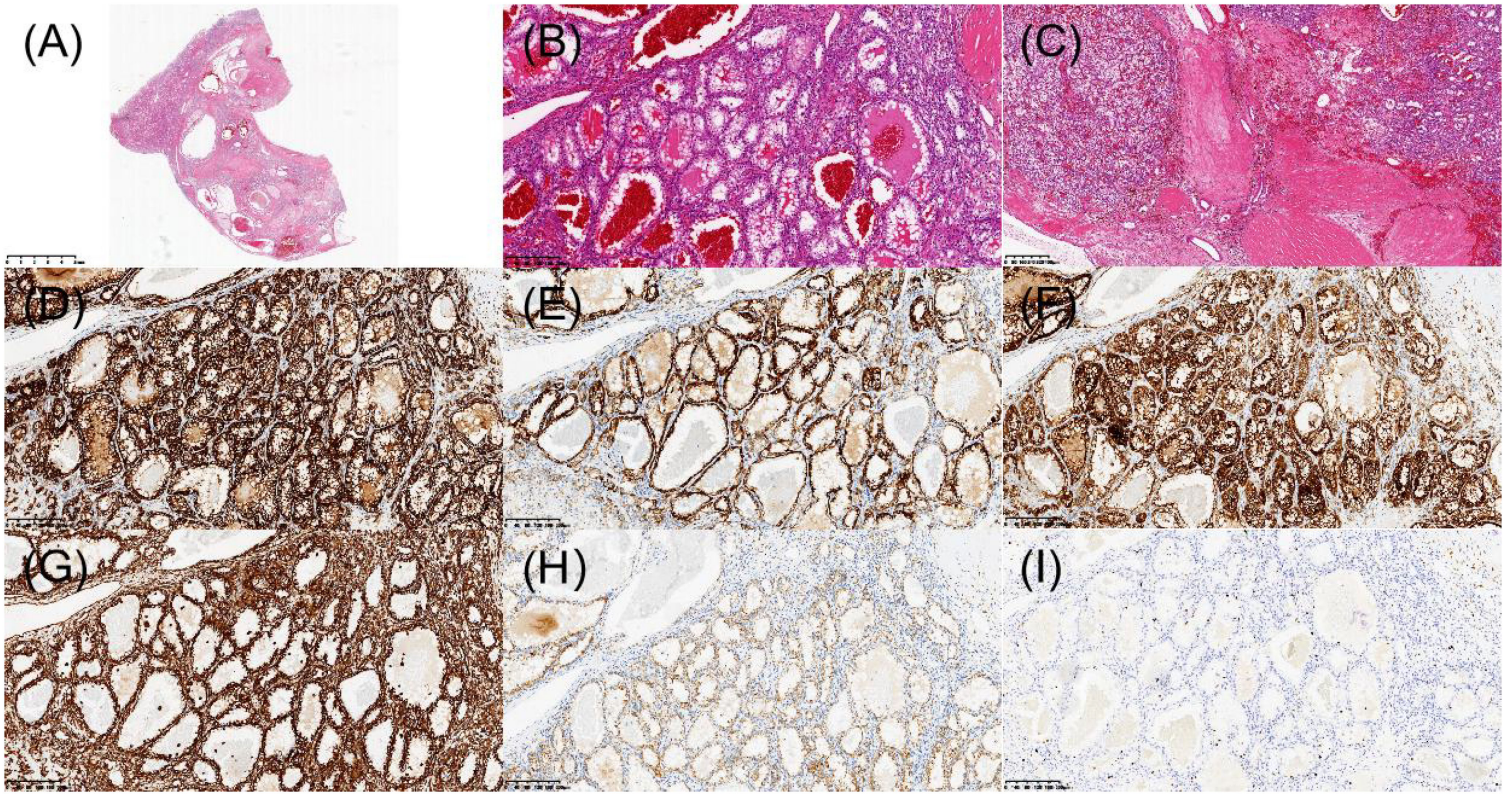
Histopathologic and immunohistochemical features of renal cell carcinoma with fibromyomatous stroma in the left kidney. **(A)** H&E staining revealed a well-demarcated tumor nodule under low-power microscopy. **(B)** Tumor cells with clear cytoplasm arranged in nests/alveolar patterns. **(C)** Prominent fibromyomatous stroma. **(D)** Immunohistochemical staining demonstrated diffuse membranous positivity for CA IX. **(E)** Focal CK7 immunoreactivity. **(F)** CD10 was positive. **(G)** Vimentin was positive. **(H)** AMACR showed abnormal membranous staining pattern (interpreted as negative). **(I)** Ki-67 was approximately 3%.

**Figure 6:**
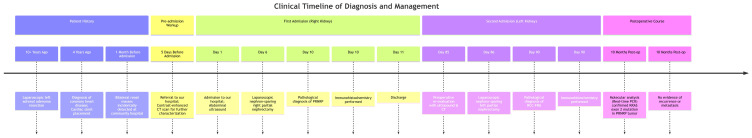
Clinical timeline of diagnosis and management.

The patient underwent laparoscopic nephron-sparing bilateral partial nephrectomy. Gross examination of the right kidney revealed a gray-yellow tissue mass measuring 4.5 × 3 × 2.5 cm. Upon sectioning, a well-circumscribed nodule with a capsule was identified, displaying a gray-red, soft, solid cut surface with a fine papillary texture. The surgical margin was inked, and the entire lesion was submitted for histologic evaluation. Pathology identified a PRNRP in the right kidney (**Figures 3A–C**), with immunohistochemistry showing positivity for CK, CK7, GATA3, 34βE12, FH, and SDHB, partial positive for CK20, TFE-3, and Vim, while AMACR, CA IX, CD10, RCC, and HMB-45 were negative, and a Ki-67 proliferation index of approximately 2% (**Figures 3D–I**). Analysis of somatic hotspot mutations across exons 2 to 4 of the KRAS and NRAS genes, along with the BRAF V600 mutation, was conducted via real-time fluorescent quantitative PCR. This analysis demonstrated a mutation in KRAS Exon 2, while no corresponding mutations were detected in NRAS or at the BRAF V600 locus (**Figure 4**). Gross examination of the left kidney specimen showed a gray-red tissue mass measuring 3.5 × 3.0 × 2.8 cm, containing a nodule measuring 2.6 × 2.0 × 2.0 cm. The cut surface was gray-white to gray-red, solid, and moderately firm, with a relatively clear boundary from the surrounding renal parenchyma. Focal microcystic changes were noted. The entire nodule was submitted for pathological assessment, which established the diagnosis of RCC-FMS (**Figures 5A–C**), immunohistochemically positive for CK, Vim, PAX-8, RCC, CA IX, CD10, SDHB, and FH, partial positive for CK7 and TFE-3, negative for 34βE12, AMACR, and CD117, with a Ki-67 proliferation index of approximately 3% (**Figures 5D–I**). During the 10-month follow-up postoperatively, no recurrence or metastasis was reported (**Figure 6**).

## 3 Discussion

The present case of bilateral synchronous renal tumors, comprising PRNRP in the right kidney and RCC-FMS in the left kidney, represents an exceptionally rare clinical scenario. This unique combination underscores the diagnostic challenges posed by rare renal neoplasms and highlights the critical role of multimodal imaging, histopathology, and multidisciplinary collaboration in achieving accurate diagnosis and tailored management.

### 3.1 Imaging and diagnostic challenges

The imaging features observed in this case align with previously reported characteristics of PRNRP and RCC-FMS but also illustrate the complexities of differentiating these entities from more common renal tumors. The right kidney mass exhibited typical PRNRP findings, including small size, exophytic growth, heterogeneous enhancement, and cystic components with high pre-contrast attenuation (4, 5). These features, while suggestive of PRNRP, overlap with other hypovascular tumors such as fat-poor angiomyolipoma or papillary renal cell carcinoma (6, 7). The left kidney mass demonstrated the hallmark stromal heterogeneity of RCC-FMS, with mixed attenuation on CT and peripheral/septal enhancement, mimicking cystic or necrotic changes seen in clear cell RCC (8, 9). The absence of macroscopic fat helped exclude AML, while the lack of marked hypervascularity distinguished it from conventional clear cell RCC.

The coexistence of these two distinct tumors in a single patient further complicated the preoperative diagnosis (10). PRNRP’s indolent behavior and RCC-FMS’s variable clinical course necessitated careful imaging-pathologic correlation to avoid misclassification and overtreatment. This case emphasizes the importance of considering rare tumor subtypes in the differential diagnosis of renal masses, particularly when imaging features deviate from typical patterns.

### 3.2 Pathological and molecular correlations

Histopathological and immunohistochemical analyses were instrumental in establishing the definitive diagnoses of both tumors. The right renal tumor exhibited the classic morphological features of PRNRP, including papillary architecture, eosinophilic cytoplasm, and the pathognomonic apical nuclear polarity. Immunohistochemically, the tumor demonstrated a characteristic profile with strong and diffuse positivity for CK7 and GATA3, focal positivity for CK20 and TFE3, and negativity for AMACR, CA IX, and CD10. The tabulated data from 385 cases revealed that PRNRP is defined by near-constant expression of GATA3 (98.96%), CK7 (97.18%), and EMA (100%), while typically showing low rates of positivity for AMACR (41.05%), CD10 (29.83%), and Vimentin (11.92%). The Ki-67 proliferation index was low at approximately 2%. Furthermore, molecular analysis confirmed a KRAS exon 2 mutation, which is a molecular hallmark of PRNRP, being present in 82.97% (268/323) of reported cases (**Table 1**), while no mutations were detected in NRAS or BRAF V600.

**Table 1:** Immunohistochemical markers and KRAS mutation status of the patients with PRNRP in the literature.

Year	Author	GATA3	CK7	PAX-8	AMACR(P504S)	CD10	Vimentin	CD117	CA IX	L1CAM	34βE12	EMA	CK20	TFE3	TFEB	SDHB	FH	KRAS mutation
2020	Zhou et al. (11)	(7/7)	(7/7)	N/A	(7/7)	(7/7)	(0/7)	(0/7)	N/A	N/A	N/A	(7/7)	N/A	N/A	N/A	N/A	N/A	N/A
2022	Chen et al. (7)	(1/1)	(1/1)	N/A	(0/1)	(1/1)	N/A	(0/1)	(0/1)	N/A	(1/1)	N/A	N/A	N/A	N/A	N/A	N/A	N/A
2022	Al-Obaidy et al. (12)	(47/47)	N/A	N/A	(12/46)	N/A	(0/44)	N/A	(0/1)	(34/38)	N/A	N/A	N/A	N/A	N/A	N/A	N/A	(29/50)
2022	Yang et al. (13)	(11/11)	(11/11)	(11/11)	(4/11)	(1/11)	(0/11)	(0/11)	(0/11)	N/A	N/A	(11/11)	N/A	(0/11)	N/A	N/A	N/A	(9/9)
2022	Wang et al. (14)	(5/5)	(14/14)	N/A	(0/12)	(2/9)	(2/7)	(0/3)	N/A	N/A	N/A	(12/12)	N/A	N/A	N/A	N/A	N/A	(4/10)
2022	Shen et al. (15)	(16/16)	(16/16)	(16/16)	(11/16)	(3/16)	(1/16)	(0/16)	(0/16)	N/A	(12/16)	N/A	N/A	N/A	N/A	N/A	N/A	(13/14)
2022	Zuo et al. (16)	(2/2)	(2/2)	(0/2)	(0/2)	(0/2)	(0/2)	(0/2)	(0/2)	N/A	(1/1)	(2/2)	N/A	(0/2)	N/A	N/A	N/A	N/A
2022	Liu et al. (17)	(20/20)	(20/20)	N/A	N/A	N/A	N/A	(0/20)	N/A	N/A	N/A	N/A	N/A	N/A	N/A	N/A	N/A	(17/18)
2022	Wei et al. (18)	(68/68)	(87/88)	N/A	(50/88)	(27/88)	(8/72)	(1/33)	N/A	N/A	N/A	N/A	N/A	N/A	N/A	N/A	N/A	(73/86)
2023	Kim et al. (19)	(43/43)	(37/42)	(43/43)	(4/43)	(21/42)	(1/43)	N/A	N/A	(43/43)	N/A	(42/42)	N/A	N/A	N/A	N/A	N/A	N/A
2023	Nova-Camacho et al. (20)	N/A	(8/8)	(8/8)	(7/8)	(2/8)	(2/8)	N/A	N/A	(8/8)	N/A	(8/8)	N/A	N/A	N/A	N/A	N/A	(5/8)
2023	Xing et al. (2)	N/A	(1/1)	N/A	(1/1)	(1/1)	(1/1)	(0/1)	(0/1)	N/A	(1/1)	(1/1)	N/A	(0/1)	N/A	(1/1)	N/A	(1/1)
2023	Satturwar et al. (21)	N/A	(1/1)	N/A	(1/1)	N/A	(0/1)	N/A	(0/1)	N/A	N/A	N/A	N/A	N/A	N/A	N/A	N/A	N/A
2023	Li et al. (22)	(1/1)	(1/1)	(1/1)	(1/1)	(0/1)	(1/1)	(0/1)	N/A	N/A	(1/1)	(1/1)	(0/1)	(0/1)	N/A	N/A	N/A	(1/1)
2024	Shao et al. (23)	(2/2)	(2/2)	(1/1)	(0/2)	N/A	(0/1)	(0/2)	(0/1)	N/A	N/A	N/A	N/A	N/A	N/A	N/A	N/A	N/A
2024	Han et al. (24)	(9/9)	(7/9)	(9/9)	N/A	(4/9)	(3/9)	(0/9)	(6/9)	N/A	N/A	N/A	N/A	(0/9)	N/A	(9/9)	(9/9)	(7/9)
2024	Nemours et al. (25)	(10/10)	(10/10)	N/A	N/A	N/A	(0/10)	N/A	N/A	(7/7)	N/A	N/A	N/A	N/A	N/A	N/A	N/A	(7/10)
2025	Sugawara et al. (26)	(0/1)	(1/1)	(1/1)	(1/1)	N/A	N/A	(0/1)	N/A	N/A	N/A	N/A	N/A	(1/1)	(1/1)	N/A	N/A	N/A
2025	Gonda et al. (27)	(2/2)	(2/2)	(1/1)	N/A	N/A	(0/1)	N/A	N/A	N/A	N/A	N/A	N/A	N/A	N/A	N/A	N/A	N/A
2025	Nithagon et al. (28)	(1/1)	(1/1)	(1/1)	N/A	N/A	N/A	(0/1)	(0/1)	N/A	N/A	N/A	(0/1)	N/A	N/A	N/A	N/A	(1/1)
2025	Xing et al. (29)	(1/1)	(1/1)	(1/1)	(1/1)	(0/1)	(0/1)	(0/1)	(0/1)	N/A	N/A	(1/1)	N/A	N/A	N/A	N/A	N/A	N/A
2025	Wang et al. (30)	(35/35)	(35/35)	N/A	(12/34)	N/A	(6/34)	N/A	N/A	N/A	N/A	N/A	N/A	N/A	N/A	N/A	N/A	(23/28)
2025	Gonzalez et al. (31)	(1/1)	(1/1)	(1/1)	(1/1)	(0/1)	(0/1)	(0/1)	(0/1)	N/A	N/A	(1/1)	N/A	N/A	N/A	N/A	N/A	N/A
2025	Wen et al. (4)	(19/20)	N/A	(7/10)	(5/25)	(5/19)	(5/21)	(0/11)	N/A	N/A	N/A	(17/17)	(7/12)	N/A	N/A	N/A	N/A	N/A
2025	Lee et al. (32)	(75/77)	(75/77)	(77/77)	(38/77)	(14/77)	(13/77)	(0/77)	(0/77)	N/A	N/A	N/A	(0/77)	(2/77)	(66/77)	(77/77)	(77/77)	(75/75)
2025	Vangapandu et al. (33)	(1/1)	(1/1)	(1/1)	(0/1)	N/A	N/A	N/A	N/A	N/A	N/A	N/A	N/A	N/A	N/A	N/A	N/A	N/A
2025	Lobo et al. (34)	(1/1)	(1/1)	(1/1)	N/A	N/A	N/A	N/A	N/A	N/A	N/A	N/A	N/A	N/A	N/A	N/A	N/A	(1/1)
2025	Wu et al. (35)	(2/2)	(1/1)	(1/2)	N/A	N/A	N/A	(0/1)	N/A	N/A	N/A	N/A	N/A	N/A	N/A	N/A	N/A	(1/1)
2025	Our case	(1/1)	(1/1)	N/A	(0/1)	(0/1)	(1/1)	(0/1)	(0/1)	N/A	(1/1)	N/A	(1/1)	(1/1)	N/A	(1/1)	(1/1)	(1/1)
	Tatol (N, %)	(381/385) 98.96%	(345/355) 97.18%	(181/187) 96.79%	(156/380) 41.05%	(88/295) 29.83%	(44/369) 11.92%	(1/200) 0.50%	(6/124) 4.84%	(92/96) 95.83%	(17/21) 80.95%	(103/103) 100.00%	(8/92) 8.70%	(4/103) 3.88%	(67/78) 85.90%	(88/88) 100.00%	(87/87) 100.00%	(268/323) 82.97%

N/A, not available.

In contrast, the left renal tumor was diagnosed as RCC-FMS, histologically characterized by nests of clear cells embedded within a prominent fibromyomatous stroma. Its immunohistochemical profile was distinct, showing positivity for PAX-8, CA IX, and CD10, partial positivity for CK7, and negativity for AMACR and CD117. The Ki-67 index was also low at approximately 3%. Although specific molecular testing for mTOR pathway genes was not performed in our case, the existing literature indicates frequent alterations in the TSC/MTOR pathway in RCC-FMS (**Table 2**), which is characterized by frequent expression of CK7 (100%), CD10 (87.5%), Vimentin (100%), and CA IX (100%) in the limited number of cases with available data.

**Table 2:** Immunohistochemical markers and gene mutation status of the patients with RCCFMS in the literature.

Year	Author	GATA3	CK7	PAX-8	AMACR(P504S)	CD10	Vimentin	CD117	CA IX	L1CAM	34βE12	EMA	CK20	TFE3	TFEB	SDHB	FH	Gene mutation
2022	Haouane et al. (9)	N/A	(1/1)	N/A	N/A	(0/1)	(1/1)	N/A	N/A	N/A	N/A	N/A	N/A	N/A	N/A	N/A	N/A	N/A
2024	Bava et al. (2)	(2/2)	(2/2)	N/A	(0/2)	(2/2)	N/A	N/A	(2/2)	N/A	N/A	N/A	N/A	N/A	N/A	N/A	N/A	mTOR(2/2)
2024	Liu et al. (36)	(0/1)	(1/1)	(1/1)	N/A	(1/1)	(1/1)	(0/1)	(1/1)	N/A	N/A	N/A	(1/1)	(0/1)	(0/1)	(1/1)	(1/1)	ASXL1(1/1)
2025	Baranova et al. (37)	N/A	(3/3)	N/A	N/A	(3/3)	(3/3)	N/A	(3/3)	N/A	N/A	N/A	N/A	N/A	N/A	N/A	N/A	TSC1(3/3)
2025	Our case	N/A	(1/1)	(1/1)	(0/1)	(1/1)	(1/1)	(0/1)	(1/1)	N/A	(0/1)	N/A	N/A	(1/1)	N/A	(1/1)	(1/1)	N/A
	Tatol (N, %)	(2/3) 66.67%	(8/8) 100.00%	(2/2) 100.00%	(0/3) 0.00%	(7/8) 87.50%	(6/6) 100.00%	(0/2) 0.00%	(7/7) 100.00%	N/A	(0/1) 0.00%	N/A	(1/1) 100.00%	(1/2) 50.00%	(0/1) 0.00%	(2/2) 100.00%	(2/2) 100.00%	(6/6) NA

N/A, not available.

While PRNRP, RCCFMS, and adrenal adenomas are distinct entities, their co-occurrence in this patient—who also has a history of an adrenal adenoma surgically removed some time ago—raises the possibility of underlying connections, though the temporal distance may make a direct relationship less certain. Several hypotheses could be considered, such as a shared embryonic origin where localized developmental anomalies or dysregulated signaling pathways like Wnt/β-catenin might simultaneously affect adrenal and renal tissues (38). Alternatively, a paracrine influence from the adrenal adenoma, potentially secreting unrecognized growth factors such as IGF or VEGF, could foster a microenvironment conducive to renal tumor development (39). A common genetic susceptibility, whether germline or somatic, might also lower the threshold for tumor formation in both organs (40). Nevertheless, the possibility of coincidence cannot be ruled out.

The concurrent diagnosis of a KRAS-driven PRNRP and a TSC/mTOR pathway-associated RCC-FMS, accompanied by an adrenal adenoma, forms a distinctive clinical scenario. This rare constellation of tumors raises important questions regarding potential common pathogenic mechanisms. Although their well-defined molecular profiles confirm these as separate entities, their simultaneous emergence points toward a possible shared systemic predisposition. Comprehensive molecular analysis may determine whether their coexistence signifies a meaningful pathogenic link or a remarkable coincidence, potentially yielding new insights into multi-organ tumorigenesis and enriching our understanding of such complex presentations.

### 3.3 Clinical implications and management

The indolent behavior of PRNRP and the generally favorable prognosis of RCC-FMS guided the decision for nephron-sparing surgery, which is curative in most cases. This approach aligns with current recommendations for small, localized renal tumors with low malignant potential.

For PRNRP, the primary treatment is surgical resection. Partial nephrectomy is the preferred approach for most small tumors (typically <4 cm), as it completely removes the tumor while preserving healthy renal tissue and minimizing the risk of postoperative renal insufficiency (29, 2). In cases of larger or more complex tumors, or when partial nephrectomy is not feasible, radical nephrectomy may be considered. For elderly patients, those with significant comorbidities, or limited life expectancy, active surveillance with regular imaging may be appropriate, especially for small, slow-growing tumors. Ablation therapies (e.g., radiofrequency or cryoablation) represent alternatives for patients who are unfit for or decline surgery (31). For advanced or metastatic PRNRP, targeted therapy and immunotherapy may be considered, although specific agents targeting common mutations like KRAS are not yet established.

For RCC-FMS, surgical resection remains the primary and often curative treatment. Partial nephrectomy is suitable for smaller, favorably located tumors, aligning with its typically indolent course, while radical nephrectomy may be required for larger or complex lesions (9). Active surveillance is a viable option for small tumors, elderly patients, or those with significant comorbidities, particularly when biopsy confirms low-grade, indolent behavior. Given the association of RCC-FMS with alterations in the TSC/MTOR pathway, mTOR inhibitors (e.g., everolimus, temsirolimus) represent a potential targeted therapeutic option for advanced, metastatic, or inoperable cases, though clinical data remain limited (2). Accurate diagnosis is crucial, as RCC-FMS must be distinguished from other renal cell carcinomas (e.g., clear cell RCC, clear cell papillary RCC, ELOC-mutated RCC) with different management strategies and prognoses.

However, the presence of bilateral synchronous tumors necessitated individualized risk stratification: PRNRP’s negligible metastatic risk justified conservative resection, while RCC-FMS’s rare aggressive potential warranted close surveillance. This case also highlights the importance of multidisciplinary collaboration. Radiologists, urologists, and pathologists must work together to integrate imaging findings, histopathology, and molecular data to optimize diagnostic accuracy and treatment planning. For instance, intraoperative frozen section analysis could aid in confirming tumor margins and subtypes, ensuring appropriate surgical extent.

### 3.4 Limitations and future directions

The rarity of these tumors limits the generalizability of findings, and the absence of long-term follow-up precludes definitive conclusions about outcomes. Prospective multicenter studies and larger cohorts are needed to refine diagnostic criteria and establish evidence-based management guidelines. Advanced imaging techniques, such as multiparametric MRI or radiomics, may further improve preoperative differentiation of rare renal neoplasms.

## 4 Conclusion

This case represents the first documented coexistence of PRNRP and RCC-FMS as bilateral synchronous renal tumors. It underscores the diagnostic challenges posed by rare renal neoplasms and the necessity of a multidisciplinary approach to achieve accurate classification and tailored therapy. Radiologists and urologists should maintain a high index of suspicion for rare tumor subtypes when encountering renal masses with atypical imaging features, ensuring optimal patient outcomes through precision medicine. Future research should focus on elucidating the molecular mechanisms underlying such rare synchronous presentations to guide personalized management strategies.

## Data Availability

The original contributions presented in the study are included in the article/**Supplementary Material**. Further inquiries can be directed to the corresponding author.
