# Supplementary figures and images for: Bilateral synchronous papillary renal neoplasm with reverse polarity and renal cell carcinoma with fibromyomatous stroma: a case report and review of the literature
Source: Front Oncol. 2025 Nov 24;15:1668258. doi: 10.3389/fonc.2025.1668258 (PMC12683331; doi:10.3389/fonc.2025.1668258)

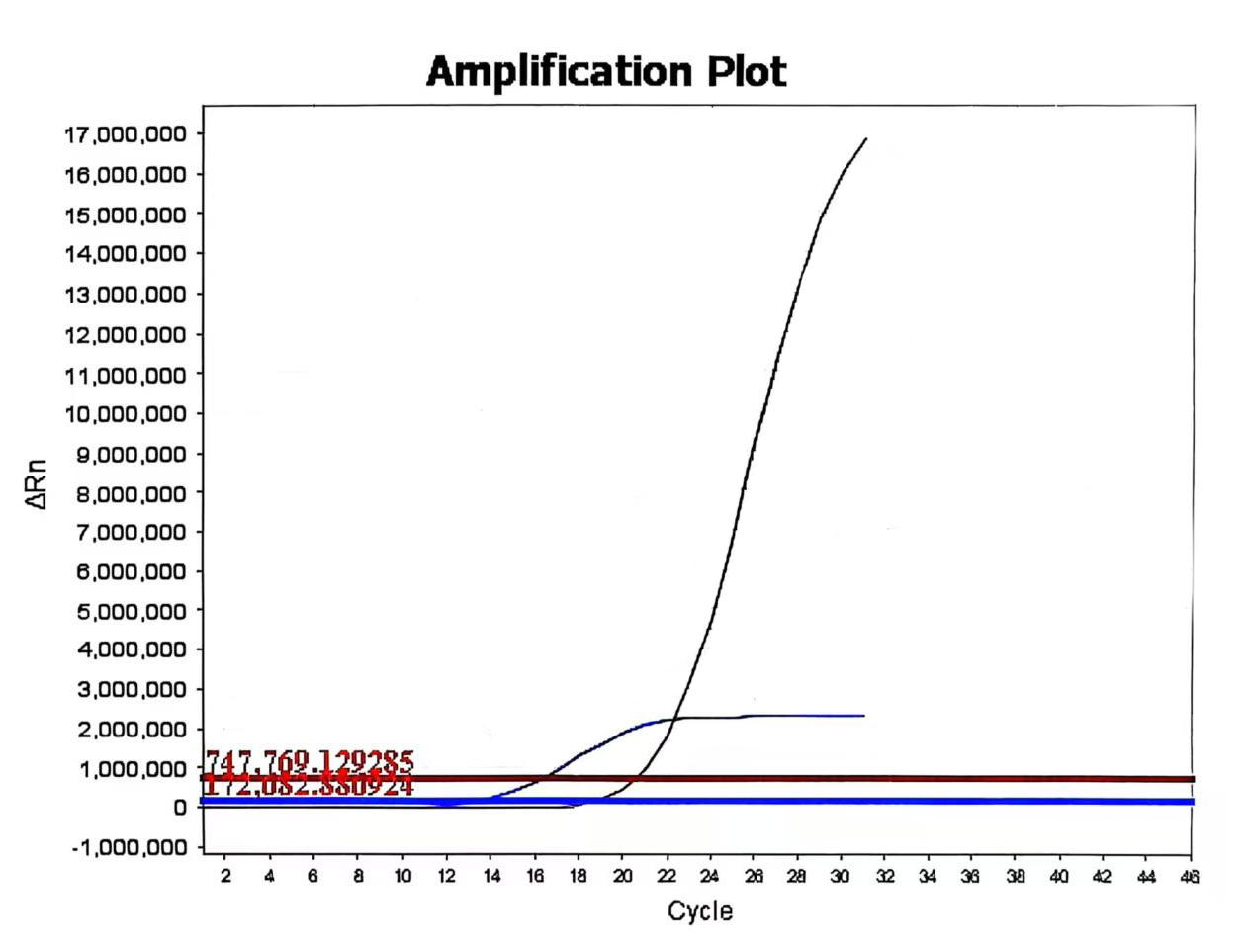

Supplement: Supplementary file 1 [file Image1.tif]

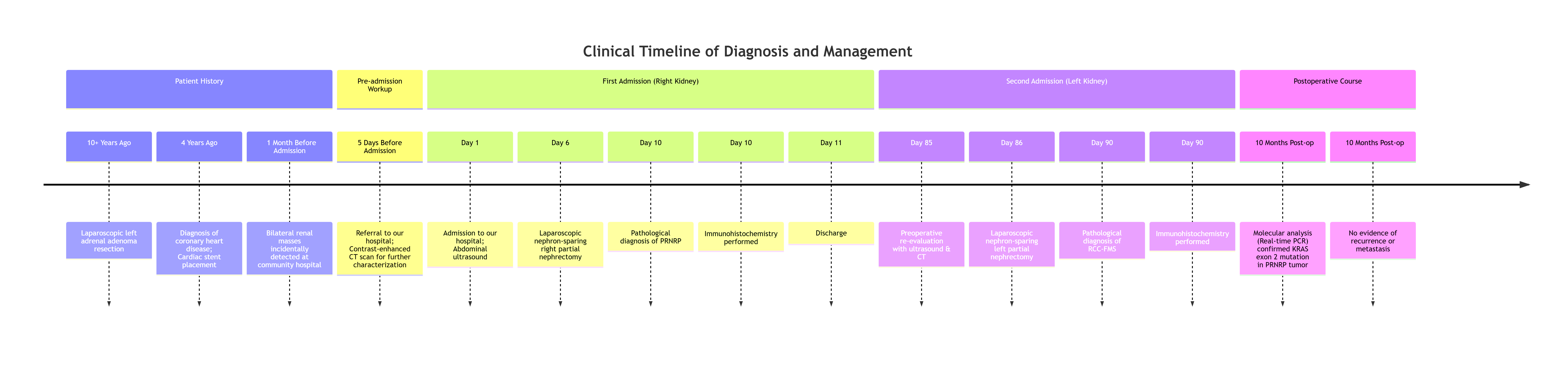

Supplement: Supplementary file 2 [file Image2.tif]
